# Linear accelerator-based stereotactic radiosurgery in 140 brain metastases from malignant melanoma

**DOI:** 10.1186/s12885-015-1517-1

**Published:** 2015-07-23

**Authors:** Henrik Hauswald, Alina Stenke, Jürgen Debus, Stephanie E. Combs

**Affiliations:** Department of Radiation Oncology, Heidelberg University Hospital, INF 400, 69120 Heidelberg, Germany

**Keywords:** Malignant melanoma, Brain metastases, SRS, Stereotactic radiosurgery, Radiotherapy

## Abstract

**Background:**

To retrospectively access outcome and prognostic parameters of linear accelerator-based stereotactic radiosurgery in brain metastases from malignant melanoma.

**Methods:**

Between 1990 and 2011 140 brain metastases in 84 patients with malignant melanoma (median age 56 years) were treated with stereotactic radiosurgery. At initial stereotactic radiosurgery 48 % of patients showed extracerebral control. The median count of brain metastases in a single patient was 1, the median diameter was 12 mm. The median dose applied was 20 Gy/80 % isodose enclosing.

**Results:**

The median follow-up was 7 months and the median overall survival 9 months. The 6-, 12- and 24 month overall survival rates were 71 %, 39 % and 25 % respectively. Cerebral follow-up imaging showed complete remission in 20 brain metastases, partial remission in 39 brain metastases, stable disease in 54 brain metastases, progressive disease in 24 brain metastases and pseudo-progression in 3 brain metastases. Median intracerebral control was 5.3 months and the 6- and 12-month intracerebral progression-free survival rates 48 % and 38 %, respectively. Upon univariate analysis, extracerebral control (log-rank, p < 0.001), the response to stereotactic radiosurgery (log-rank, p < 0.001), the number of brain metastases (log-rank, p = 0.007), the recursive partitioning analysis class (log-rank, p = 0.027) and the diagnosis-specific graded prognostic assessment score (log-rank, p = 0.011) were prognostic for overall survival. The most common clinical side effect was headache common toxicity criteria grade I. The most common radiological finding during follow-up was localized edema within the stereotactic radiosurgery high dose region.

**Conclusion:**

Stereotactic radiosurgery is a well-tolerated and effective treatment option for brain metastases in malignant melanoma and was able to achieve local remissions in several cases. Furthermore, especially patients with controlled extracerebral disease and a low count of brain metastases seem to benefit from this treatment modality. Prospective trials analysing the effects of combined stereotactic radiosurgery and new systemic agents are warranted.

## Background

The predicted 2012 standardized disease rate for malignant melanoma (MM) in Germany for women is 15.6 and for men 16.9 per 100.000 persons, respectively [1]. Even though incidence rates worldwide have increased over the past decades, recent developments indicate stabilization in some high-risk countries [2]. Risk factors for the development of brain metastases (BM) are for example positive sentinel lymph nodes and primary tumor ulceration [3, 4]. Unfortunately, the prognosis with BM from MM is poor and varies between a median overall survival of 3.5 months after whole brain radiotherapy (WBRT) in case of multiple BM [5] and an actuarial median survival of 10.6 months after stereotactic radiosurgery (SRS) of single BM [6]. Another well-established approach is the resection of BM [7, 8] while upcoming systemic therapies have not shown to be adequately effective in BM from MM [9]. Prognostic factors include the Radiation Therapy Oncology Group recursive partitioning analysis (RTOG-RPA) class [10], diagnosis-specific Graded Prognostic Assessment (ds-GPA) score [11] and serum-lactate dehydrogenase (LDH) values [12]. This retrospective analysis was focused on patients with BM from MM treated with SRS to evaluate outcome and SRS-related side effects.

## Methods

### Patient characteristics

Between 1990 and 2011 181 patients with BM from MM were treated with linear accelerator (Linac)-based SRS at the Department of Radiation Oncology at the University Hospital of Heidelberg. Eighty-four patients with available imaging follow-up were included in this analysis; the remaining 97 patients without imaging follow-up were excluded from analysis. At initial SRS 48 % of patients showed extracerebral control. The median count of BM in a single patient was 1 and the median diameter 12 mm. Thirty-eight patients had > 1 BM treated with SRS. LDH levels were not evaluated on a regular basis. Further patient characteristics are found in Table 1.

**Table 1:** Patient characteristics

Patient characteristics	%	[n]
Gender		
ᅟMale	55	46
ᅟFemale	45	38
Age at initial SRS		
ᅟMedian 56 years (range, 19–94)		
Clark level		
ᅟII	2	2
ᅟIII	5	4
ᅟIV	44	37
ᅟV	4	3
ᅟn. a.	45	38
Histopathology		
ᅟALM	5	4
ᅟAMM	5	4
ᅟNM	18	15
ᅟSSM	21	18
ᅟn. a.	52	43
Extracerebral tumor control		
ᅟUncontrolled	52	44
ᅟControlled	48	40
RPA class		
ᅟ1	11	9
ᅟ2	87	73
ᅟ3	2	2
DS-GPA score		
ᅟ2	15	13
ᅟ3	43	36
ᅟ4	42	35
Symptomatic before SRS		
ᅟNo	67	56
ᅟYes	33	28
Number of BM at initial SRS		
ᅟ1	58	49
ᅟ2–3	37	31
ᅟ≥4	5	4
Size of BM		
ᅟMedian 12 mm (range, 2–36 mm)		
Localization of BM at initial SRS		
ᅟInfratentorial	8	7
ᅟSupratentorial	85	71
ᅟBoth	7	6

### Radiotherapy and follow-up

SRS applied a median dose of 20 Gy on the enclosing 80 % isodose. SRS was performed Linac-based using 6-mega electron volt (MeV) photon beams with either a round collimator or individually shaped by a micro-multileaf collimator. Head fixation was ensured by Scotchcast-masks. Patients were regularly followed by clinical examinations and imaging procedures as computer tomography (CT) or magnetic resonance imaging (MRI). Salvage treatments consisted of whole brain radiotherapy, surgical resection of the BM, and chemo- or more recently immunotherapy.

### Evaluation and statistics

The toxicity was graded according to the Common Toxicity Criteria for Adverse Events (CTCAE Version 4). The Kaplan-Meier survival analysis was used to estimate survival curves. Univariate analysis included age (>/< median age), gender, localization of the BM (infra- vs. supratentorial), number of BM (total and grouped 1 vs. 2–3 vs. >3), response to SRS (remission (including complete and partial remission) vs. stable disease vs. progressive disease), size of BM (>/< median), extracerebral tumor control (yes vs. no), Karnofsky performance score (90–100 vs. 70–80 vs. <70), RPA (1 vs. 2 vs. 3), ds-GPA (2 vs. 3 vs. 4), WBRT during follow-up (yes vs. no) and clinical symptoms prior to SRS (yes vs. no). Multivariate analysis was performed with the Cox-regression model (backwards stepwise, p out >0.1). Multivariate analysis included the significant factors from univariate analysis: extracerebral control, ds-GPA score, RPA class, number of BM and response to SRS. Significance was defined as p < 0.05. Correlation of the treatment response after SRS in patients with 2 or more BM treated with SRS was analyzed using Spearman correlation coefficient. All time estimates began with the date of SRS. The statistical analyses were carried out using SPSS (SPSS Inc., Chicago, IL, USA). Informed consent was obtained. The study was approved by the Ethics Committee of the University of Heidelberg (S-004/2012).

## Results

### Outcome

**Fig. 1:**
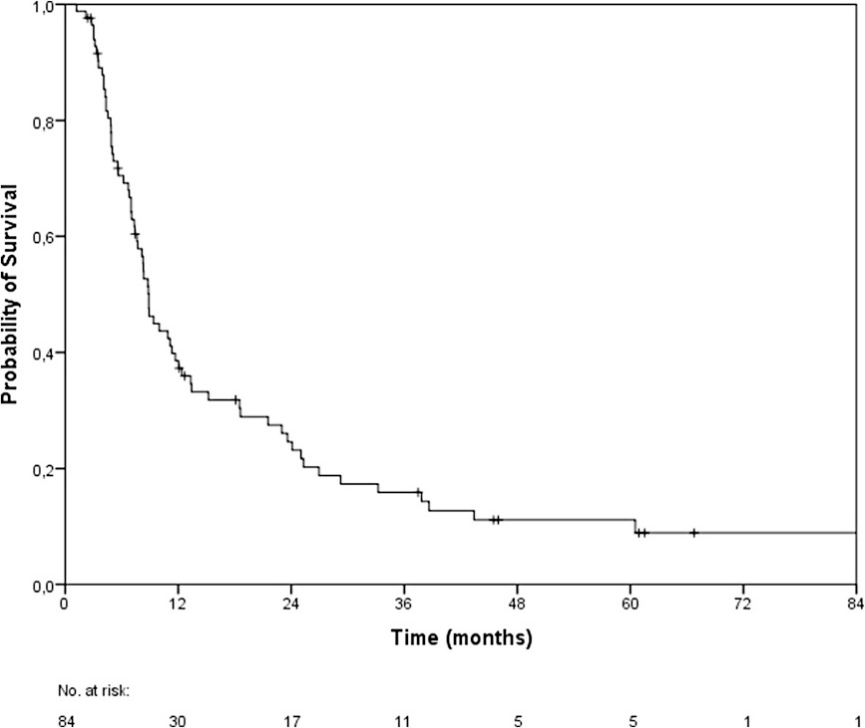
Kaplan-Meier estimation of overall survival (n = 84)

**Fig. 2:**
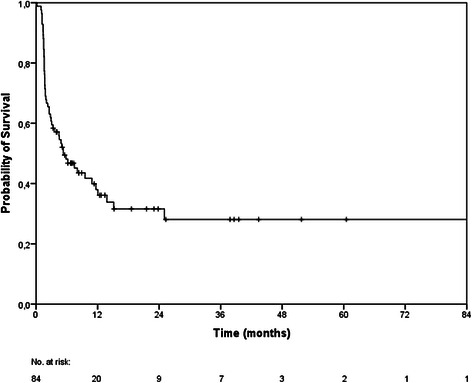
Kaplan-Meier estimation of intracerebral progression-free survival

The median OS was 9 months (95 % CI 8–10 months). The 6-, 12- and 24-months OS rates were 71 %, 39 % and 25 % (Fig. 1). At the last follow-up examination in July 2014, 11 patients were still alive. Causes of death were documented in 6 patients only: intracerebral progression in 4 patients and peritoneal carcinomatosis as well as pulmonary embolism in 1 patient each. The median follow-up time was 7 months (range, 0.2–199.2 months). Cerebral follow-up imaging showed a complete remission (CR) in 20 BM, a partial remission (PR) in 39 BM, stable disease (SD) in 54 BM, progressive disease (PD) in 24 BM and a histopathologically proven pseudo-progression in 3 BM. The median intracerebral control time was 5.3 months resulting in 6- and 12-months intracerebral progression-free survival rates of 48 % and 38 % (Fig. 2).

### Prognostic factors

**Fig. 3:**
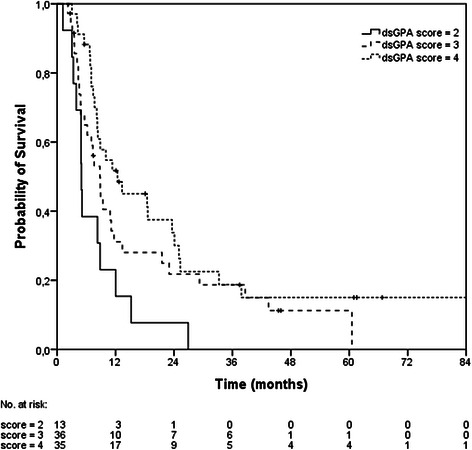
Kaplan-Meier estimation of overall survival according to the ds-GPA score (score 2, n = 13; score 3, n = 36; score 4, n = 35)

The results of the uni- and multivariate analyses are presented in Table 2. In univariate analyses, extracerebral tumor control (p < 0.001, uncontrolled 6.8 months vs. controlled 12.4 months), response to SRS (p < 0.001, progressive disease 4.3 months vs. stable disease 8.3 months vs. remission 13.3 months), number of BM (linear p = 0.007; grouped p = 0.005, n > 3 8.3 months vs. n = 2–3 5 months vs. n = 1 12.4 months), RPA class (p = 0.027, class 1 37.8 months vs. class 2 8.3 months versus class 3 3.3 months) and ds-GPA (p = 0.011, score 2 5 months vs. score 3 8.8 months vs. score 4 12.4 months, Fig. 3) were prognostic for overall survival (OS). In multivariate analysis extracerebral tumor control (p < 0.001), response to SRS (p < 0.001) and the grouped number of BM (p = 0.006) were prognostic. In patients with 2 or more BM treated with SRS the treatment response after SRS correlated significantly (Spearman correlation coefficient 0.684).

**Table 2:** Uni- and multivariate analyses

Univariate analysis (log-rank)	p-value
ᅟGender (male vs. female)	0.587
ᅟAge (>/< median)	0.498
ᅟExtracerebral tumor control (yes vs. no)	*<0.001*
ᅟKPS (grouped 90–100 vs. 70–80 vs. <70)	0.649
ᅟRPA (1 vs. 2 vs. 3)	*0.027*
ᅟds-GPA (2 vs. 3 vs. 4)	*0.011*
ᅟWBRT (yes vs. no)	0.082
ᅟSymptoms prior to SRS (yes vs. no)	0,228
ᅟNumber of BM (grouped 1 vs. 2–3 vs. >3)	*0.005*
ᅟNumber of BM (total)	*0.007*
ᅟLocation (infra- vs. supratentorial)	0.792
ᅟResponse to SRS (remission vs. stable vs. progression)	*<0.001*
ᅟSize of BM (>/< median)	0.125
Multivariate Analysis (Cox-regression model)	p-value
ᅟExtracerebral tumor control (yes vs. no)	*<0.001*
ᅟds-GPA (2 vs. 3 vs. 4)	0.078
ᅟRPA (1 vs. 2 vs. 3)	0.208
ᅟNumber of BM (grouped 1 vs. 2–3 vs. >3)	*0.006*
ᅟResponse to SRS	*<0.001*

### Side effects

Acute side effects within the first three days after SRS were seen in 6 % (n = 5): headache CTCAE °I was reported by 3 patients, muscle weakness CTCAE °II by 1 patient and temporary worsening of pre-existing paresthesias CTCAE °I by one other patient. Acute side effects within the first 3 months were documented in 13 patients (15 %; Table 3). Late (>3 months) side effects were documented in 2 patients (Table 3).

**Table 3:** Acute and chronic adverse events according to CTCAE

Acute adverse events	% [n]
ᅟLocalized edema	
ᅟᅟ°I	5 [6]
ᅟᅟ°II	2 [2]
ᅟᅟ°III	1 [1]
ᅟHeadache	
ᅟᅟ°I	3 [4]
ᅟAlopecia	
ᅟᅟ°I	1 [1]
ᅟDizziness	
ᅟᅟ°I	1 [1]
ᅟNausea	
ᅟᅟ°I	1 [1]
ᅟIntracranial hemorrhage	
ᅟᅟ°I	1 [1]
ᅟCentral nervous system necrosis	
ᅟᅟ°I	1 [1]
Late adverse events	
ᅟLocalized edema	
ᅟᅟ°I	1 [1]
ᅟᅟ°II	1 [1]
ᅟCentral nervous system necrosis	
ᅟᅟ°I	1 [1]

## Discussion

This retrospective single-center analysis reports on possible prognostic factors, outcome and toxicity of SRS in 140 BM from MM treated between 1990 and 2011 and followed by cerebral imaging. Our intention was to help find ways to improve prognosis, morbidity and mortality in patients with BM from MM. Literature on treatment outcome is summarized in Table 4.

**Table 4:** SRS for BM from MM: overview of recent literature

	Lwu 2013	Marcus 2013	Bernard 2012	Liew 2011	Present study
Number of patients	36 (20 melanoma)	135	54	333	84
SRS technique	Gamma Knife	SRS	Cyber Knife	Gamma Knife	Linac based SRS
Median dose	21 Gy	n. r.	24 Gy/80 % isodose	18 Gy marginal dose	20 Gy/80 % isodose
Follow-up [months]	6	n. r.	5	3.8	7
Count of BM [n]	1: 15	1: 80	Median 1 (1–6)	1: 122	1: 49
		2–3: 35		2–3: 104	2–3: 31
	≥2: 21	≥4: 20		≥4: 107	≥4: 4
Response to SRS [%]					
Remission				29	42
Stable	n. r.	n. r.	n. r.	61	39
Progression				10	17
Median survival after SRS	n. r.	6.9	4	5.6	9
12 months survival [%]	n. r.	33.8 % with serum LDH < 240 15.4 % with serum LDH ≥ 240	25	24.9	39
24 months survival [%]	n.r.	n.r.	n.r.	9.5	25

Liew et al. reported in 2011 on 333 consecutive patients treated with Gamma Knife SRS for BM from MM [13]. The median follow-up was 3.8 months and the median survival 5.6 months. In the analysis published by Bernard et al. encompassing 54 patients with BM from MM, the median survival after SRS for intact BM (n = 34) was 4 months, compared to 13 months after prior resection (n = 20) of the BM [14]. Recently, Marcus and co-workers reported a median OS of 6.9 months in 135 patients treated with SRS for BM from MM [12]. In comparison our results showing a median OS of 9 months and 12- and 24 months OS rates of 39 % and 25 %, respectively, are superior to these prior reports. This difference might be explained by a selection bias for follow-up imaging in our analysis or different approaches in systemic therapy in case of tumor progression.

With respect to local control Liew et al. reported a median progression-free survival of 30 months with progression-free survival rates of 63 % at 12 months and 57 % at 24 months after SRS [13]. Follow-up imaging in 259 patients with 1226 BM showed CR in 6 %, PR in 23 %, SD in 61 % and PD in 10 %. In the cohort of Lwu et al. on 36 patients treated with Gamma Knife SRS (median prescription dose 21 Gy) for BM, 20 patients suffered from melanoma [15]. The local control at 12 months was 75 % for melanoma patients. In our cohort comparable response rates were observed. The diagnosis of pseudo-progression in all of our 3 cases was based on histopathological examination following surgical resection of the lesion.

Regarding the number of BM, Liew et al. reported patients suffering from single BM to have median survival of 8.2 months, compared to 4.1 months with multiple BM [13]. This prognostic difference is in accordance with our results, which showed significantly shorter survival times with increasing number of BM. On the other hand, in the smaller patient group of Marcus et al., the number of BM had no significant impact on survival [12]. In the cohort of Bernard et al., an increasing number of BM showed a trend towards shorter survival [14]. This difference might be due to selection bias caused by different treatment approaches in the different clinics.

The adverse events documented in our cohort were comparable to previous reports. One case of focal alopecia was due to superficial location of the BM and not unexpected when reviewing the treatment plan and dose distribution. One case with imaging diagnosis of central nervous system necrosis grade I was followed without intervention. Silk et al. reported on patients treated with ipilimumab and radiation therapy for BM from MM. In their comparison group intratumoral haemorrhage happened in 12.5 % of cases, radiation necrosis in 3 cases [16]. In an analysis by Kondziolka the haemorrhage rate was up to 50 % in BM from MM [17]. The diagnosis of pseudo-progression of cerebral lesions following radiation treatment is challenging. Hoefnagels et al. as well as Mitsuya et al. recommended perfusion MRI (PWI) to differentiate between progression and pseudo-progression [18, 19]. Recently Wiggenraad et al. analysed 10 patients with pseudo-progression following SRS of BM and concluded that consecutive MRIs using cine-loops may improve understanding of pseudo-progression [20]. However, in their analysis on PWI, magnetic resonance spectroscopy and amino-acid positron emission tomography Kickingereder et al. concluded that technical limitations were problematic, comparative studies warranted and stereotactic biopsies on structural MRI highly reliable to differentiate between tumor progression and radiation-induced changes [21].

However, patients with cerebrally metastasized MM have a poor prognosis. Potential for improvement might be found in the availability of new systemic therapies and the combination of those with SRS. In recent years systemic treatment options such as for example the development of cytotoxic T-lymphocyte-associated Protein 4 antibodies or BRAF inhibitors improved outcome in metastasized MM. Combined with SRS these systemic agents might provide yet further improvement, but they may also be a reason for major concern due to potential harmful interactions. A retrospective analysis by Tazi et al. suggested that survival of patients with BM from MM treated with ipilimumab combined with SRS might be comparable to those without BM [17]. Furthermore, an abscopal effect of SRS after ipilimumab has been reported prolonging the median survival to 22.4 months [22]. Silk et al. reported a five time increase in the median survival after combination of ipilimumab and SRS suggesting synergistic effects for this treatment approach [16]. Recently a study on 30 patients treated with BRAF inhibitor and Gamma knife-SRS did not show increased toxicity rates [23]. On the other hand increased skin reactions have been reported for the combination of BRAF inhibitors and radiotherapy demanding caution in combining new systemic agents and radiation treatments [24, 25].

Regarding limitations of our study, the reader should acknowledge its retrospective character and the changes in systemic treatments during its 21 year recruiting time. Prognostic markers like LDH levels or ulceration of the primary tumor were not accessible due to the retrospective character. In general, regular radiotherapeutic follow-up examinations in patients with metastasized MM could be challenging for example due to a decline in performance status caused by progressive disease, long distance to the radiation clinic or organizational difficulties in case of further treatments elsewhere. Even though all available data were carefully reviewed, a bias, for example in patient selection for MR imaging during follow-up could not be excluded. In recent years MRI capacities have increased and therefore more precise imaging information during follow-up is available than in earlier years. Furthermore, causes of death were documented in a minority of patients only. On the other hand our study has a reasonable number of treated BM and increases the available evidence for treating BM from MM with SRS. Further evaluations of prognostic markers and the immunological effects of systemic agents as ipilimumab in combination with SRS or whole brain radiation are warranted. Therefore we prepared a prospective observational trial that started recruitment recently.

## Conclusion

SRS is a well-tolerated and effective treatment option in brain metastases from malignant melanoma. Furthermore, remissions of BM could be achieved in some cases. Especially patients with controlled extracerebral disease and low count of BM seem to benefit from this treatment modality. Prospective trials analysing the effects of a treatment approach combining new systemic agents and SRS are warranted. A prospective observational study to analyse the immunologic effects of ipilimumab as well as SRS is underway.

## Abbreviations

ALM: Acral lentiginous melanoma
AMM: Amelanotic melanoma
BM: Brain metastases
CI: Confidence interval
CR: Complete remission
CT: Computer tomography
CTCAE: Common toxicity criteria for adverse events
DS-GPA: Diagnosis-specific graded prognostic assessment
Gy: Gray
KPS: Karnofsky performance score
LDH: Lactate dehydrogenase
Linac: Linear accelerator
MeV: Mega electron volt
MM: Malignant melanoma
mm: Millimeter
MRI: Magnetic resonance imaging
n. a.: Not available
NM: Nodular melanoma
n. r.: Not reported
OS: Overall survival
PD: Progressive disease
PR: Partial remission
PWI: Perfusion MRI
RPA: Recursive partitioning analysis
RTOG: Radiation Therapy Oncology Group
SD: Stable disease
SRS: Stereotactic radiosurgery
SSM: Superficial spreading melanoma
WBRT: Whole brain radiotherapy
